# Pancreatic SEC23B deficiency is sufficient to explain the perinatal lethality of germline SEC23B deficiency in mice

**DOI:** 10.1038/srep27802

**Published:** 2016-06-14

**Authors:** Rami Khoriaty, Lesley Everett, Jennifer Chase, Guojing Zhu, Mark Hoenerhoff, Brooke McKnight, Matthew P. Vasievich, Bin Zhang, Kärt Tomberg, John Williams, Ivan Maillard, David Ginsburg

**Affiliations:** 1 Department of Internal Medicine, University of Michigan, Ann Arbor, MI, USA; 2 University of Michigan Medical School, Ann Arbor, MI, USA; 3 Life Sciences Institute, University of Michigan, Ann Arbor, MI, USA; 4 In Vivo Animal Core, Unit for Laboratory Animal Medicine, University of Michigan, Ann Arbor, MI, USA; 5 College of Literature Science and the Arts, University of Michigan, Ann Arbor, MI, USA; 6 Genomic Medicine Institute, Cleveland Clinic Lerner Research Institute, Cleveland, OH, USA; 7 Department of Human Genetics, University of Michigan, Ann Arbor, MI, USA; 8 Department of Physiology, University of Michigan, Ann Arbor, MI, USA; 9 Department of Pediatrics and Communicable Diseases, University of Michigan, Ann Arbor, MI, USA; 10 Howard Hughes Medical Institute, University of Michigan, Ann Arbor, MI, USA

## Abstract

In humans, loss of function mutations in *SEC23B* result in Congenital Dyserythropoietic Anemia type II (CDAII), a disease limited to defective erythroid development. Patients with two nonsense *SEC23B* mutations have not been reported, suggesting that complete SEC23B deficiency might be lethal. We previously reported that SEC23B-deficient mice die perinatally, exhibiting massive pancreatic degeneration and that mice with hematopoietic SEC23B deficiency do not exhibit CDAII. We now show that SEC23B deficiency restricted to the pancreas is sufficient to explain the lethality observed in mice with global SEC23B-deficiency. Immunohistochemical stains demonstrate an acinar cell defect but normal islet cells. Mammalian genomes contain two *Sec23* paralogs, *Sec23A* and *Sec23B*. The encoded proteins share ~85% amino acid sequence identity. We generate mice with pancreatic SEC23A deficiency and demonstrate that these mice survive normally, exhibiting normal pancreatic weights and histology. Taken together, these data demonstrate that SEC23B but not SEC23A is essential for murine pancreatic development. We also demonstrate that two BAC transgenes spanning *Sec23b* rescue the lethality of mice homozygous for a *Sec23b* gene trap allele, excluding a passenger gene mutation as the cause of the pancreatic lethality, and indicating that the regulatory elements critical for *Sec23b* pancreatic function reside within the BAC transgenes.

Approximately 8,000 mammalian proteins are transported from the endoplasmic reticulum (ER) to the Golgi apparatus via coat protein complex II (COPII)-coated vesicles^1^,^2^, before reaching their final destination in the plasma membrane, intracellular organelles, or extracellular space. The COPII coat is composed of 5 core components, SAR1, SEC23, SEC24, SEC13 and SEC31^2^,^3^,^4^. COPII coat assembly begins on the ER cytosolic surface when the guanine nucleotide exchange factor SEC12 converts GDP-bound SAR1 to GTP-bound SAR1^5^,^6^. SAR1-GTP recruits the SEC23-SEC24 heterodimer to the ER surface through direct binding to SEC23^7^,^8^. SAR1-SEC23-SEC24 form the inner layer of the COPII coat. Following cargo recruitment^9^,^10^,^11^,^12^, SEC13-SEC31 heterotetramers form the outer layer of the COPII coat, facilitating budding of the COPII vesicle from the surface of the ER^13^,^14^,^15^,^16^. The COPII components are conserved throughout eukaryote evolution, but unlike yeast, mammals exhibit multiple paralogs for most subunits, including 2 *SEC23* paralogs, *SEC23A* and *SEC23B*, encoding two highly similar proteins (~85%). In humans, *SEC23A* missense mutations result in cranio-lenticulo-sutural-dysplasia (CLSD), an autosomal recessive disease characterized by skeletal abnormalities, late closure of fontanelles, dysmorphic features, and sutural cataracts^17^, while multiple loss of function *SEC23B* mutations have been identified in patients with congenital dyserythropoietic anemia type II (CDAII)^18^. CDAII is an autosomal recessive disease characterized by moderate anemia, increased bi/multi-nucleated erythroblasts in the bone marrow (BM), a double red blood cell (RBC) membrane by electron microscopy, and a faster than normal migration of the RBC membrane protein band 3 on SDS-PAGE^1^,^19^,^20^,^21^,^22^. No other non-hematologic abnormalities have been reported to result from *SEC23B* mutations, though a recent report suggested that germline heterozygous *SEC23B* variants are associated with Cowden syndrome and apparently sporadic thyroid cancer^23^. Patients with two nonsense *SEC23B* mutations have never been reported^1^,^24^, raising the possibility that complete SEC23B deficiency might be lethal.

We previously reported that mice homozygous for a *Sec23b* gene-trap allele (*Sec23b*^*gt/gt*^) die perinatally, with analysis at embryonic day 18.5 (E18.5) demonstrating degeneration of the pancreas and other professional secretory tissues^25^. Lethally irradiated mice transplanted with hematopoietic stem cells (HSC) deficient for SEC23B did not exhibit anemia or other CDAII characteristics^24^, and *Sec23b*^*gt/gt*^ HSC exhibited no competitive disadvantage at reconstituting the BM erythroid lineage. We also recently reported that mice homozygous for a *Sec23a* gene trap allele die at mid-embryogenesis, exhibiting a neural tube defect and impaired collagen secretion, reminiscent of the human phenotype^26^.

We now confirm and extend our previous findings, demonstrating that mice with erythroid specific (Epo-R Cre) and pan-hematopoietic (Vav1-Cre) *Sec23b* deletion support a normal erythroid and hematopoietic compartment. We also show that two BAC transgenes comprising *Sec23b* rescue the phenotype of *Sec23b*^*gt/gt*^ mice, ruling out a passenger gene mutation and indicating that key *Sec23b* regulatory elements reside within these BAC transgenes. Finally, we demonstrate that loss of SEC23B expression exclusively in the pancreas is sufficient to explain the lethality of mice with germline deletion of *Sec23b*, and show that pancreatic SEC23A, unlike SEC23B, is dispensable for normal murine pancreatic development.

## Results

### The key regulatory sequences required for *Sec23b* pancreatic function reside within a 127 kb spanning the *Sec23b* gene

**Figure 1:**
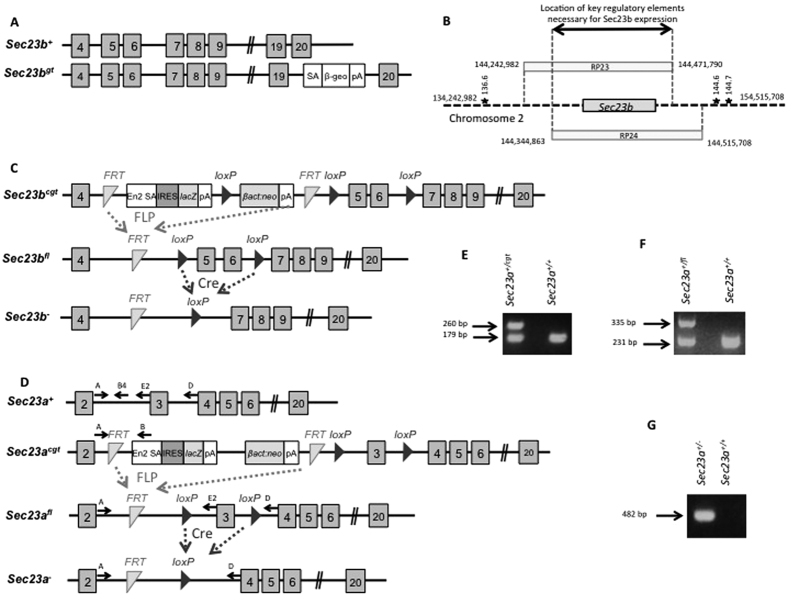
*Sec23* mutant alleles and locations of the bacterial artificial chromosome (BAC) transgenes and microsatellite markers on mouse chromosome 2. (**A**) *Sec23b*^+^: *Sec23b* wild type allele; *Sec23b*^*gt*^: the original *Sec23* gene trap allele containing a gene trap insertion in intron 19. (**B**) BAC transgenes RP23 and RP24 contain the entire Sec23 gene as well as upstream and downstream sequences as indicated. The relative locations of the microsatellites markers used for microsatellite genotyping are depicted by stars. (**C**) A second, independent, *Sec23b* conditional gene trap allele (*Sec23b*^*cgt*^) contains a gene trap insertion in intron 4. FLP mediated excision yields the conditional *Sec23b* floxed allele (*Sec23b*^*fl*^), and subsequent Cre mediated excision results in the *Sec23b* null allele (*Sec23b*^−^). (**D**) The *Sec23a* wild type allele is referred to as *Sec23a*^+^. The *Sec23a* conditional gene trap (*Sec23a*^*cgt*^) allele was crossed to a FLP mouse, resulting in the *Sec23a* floxed allele (*Sec23a*^*fl*^). Subsequent Cre mediated excision gives rise to the *Sec23a* null allele (*Sec23a*^−^). The locations of genotyping primers are depicted by arrows. (**E**) Competitive PCR assay with 1 forward primer (primer A) and 2 reverse primers (primers B and B4) yields a 179 base pair (bp) product from the wild type allele (primers A and B4), and a 260-bp product from the *Sec23a*^*cgt*^ allele (primers A and B). (**F**) PCR assay using primers A and E2 results in a 231 bp product from the wild type allele and a 335 bp product from the *Sec23b*^*fl*^ allele. (**G**) PCR assay using primers A and D results in a 482 bp product from the *Sec23b*^−^ allele. No PCR product is generated with these primers from a WT allele due to the large distance between the primers.

Mice homozygous for a *Sec23b* gene-trap allele (Fig. 1A) were previously reported to exhibit massive pancreatic degeneration with uniform perinatal lethality^25^. In contrast, pancreatic abnormalities have not been observed in SEC23B deficient humans^1^. Two independent mouse BAC transgenes (Fig. 1B) spanning the *Sec23b* gene were crossed onto the *Sec23b*^*gt*^ line. Both *Sec23b* BAC transgenes demonstrated full rescue of the *Sec23b*^*gt/gt*^ lethality, with the expected numbers of *Sec23b*^*gt/gt*^ Tg^+^ observed at weaning (Table 1). *Sec23b*^*gt/gt*^ Tg^+^ mice exhibited normal survival (observed for 300+ days), and fertility, with no apparent pancreatic or other abnormalities on routine necropsy (Supplementary Fig. 1). BAC transgene expression in the relevant tissues is inferred from the dramatic phenotype rescue. These data demonstrate that the key regulatory elements necessary for *Sec23b* pancreatic function are located within the minimum ~127 kb region shared by the two BAC transgenes (Fig. 1B).

An intercross between mice heterozygous for the *Sec23b*^−^ allele (Fig. 1C, Table 1) generated no *Sec23b*^−/−^ mice at weaning (p < 0.0001), though *Sec23b*^−/−^ mice were present at the expected frequency at E17.5 (p > 0.9) and E18.5 (p > 0.7), consistent with the previously reported perinatal lethality^25^. *Sec23b*^+/−^ mice were present at the expected numbers at weaning (Table 1), and exhibited no gross or microscopic abnormalities. E18.5 *Sec23b*^−/−^ embryos were significantly smaller than their littermates (Supplementary Fig. 2A), with massive pancreatic hypoplasia uniformly observed in the former (Supplementary Fig. 2B), as well as variable vacuolation of salivary glands, gastric pit epithelial cells, and nasal glands (Supplementary Fig. 2C).

### Loss of pancreatic SEC23B expression is sufficient to account for the perinatal mortality observed in *Sec23b* ^−/−^ mice

Pancreas-specific *Sec23b* deficient mice were generated, using the *Pdx-Cre* transgene. Out of 74 progeny generated from a *Sec23b*^+*/fl*^ *Pdx-Cre*^+^ × *Sec23b*^+/−^ cross, only 2 *Sec23b*^−*/fl*^ *Pdx-Cre*^+^ mice were observed at weaning (compared to 9/74 mice expected, p < 0.016, Table 2). Analysis of pancreas tissues from these 2 mice detected a high level of residual functional non-excised *Sec23b*^*fl*^ alleles (Supplementary Fig. 3), likely explaining their extended survival, either due to incomplete excision of *Sec23b* and/or selection of non-excised cells during pancreatic development.

Analysis of a similar cross for a second pancreas-specific Cre transgene, *p48-Cre*, resulted in 0/148 *Sec23b*^−*/fl*^ *p48-Cre*^+^ mice observed at weaning (p < 0.0001, Table 2), though *Sec23b*^−*/fl*^ *p48-Cre*^+^ mice were present at the expected numbers at E18.5 (p > 0.5, Table 2). Four mice from this cross died within 1 day of birth, all of which were genotyped as *Sec23b*^−*/fl*^ *p48-Cre*^+^ mice (Table 2).

Taken together, these data demonstrate that loss of SEC23B exclusively in the pancreas is sufficient to explain the perinatal mortality observed in mice with germline deletion of *Sec23b*.

### Pancreatic SEC23B deficiency results in loss of pancreatic acini

**Figure 2:**
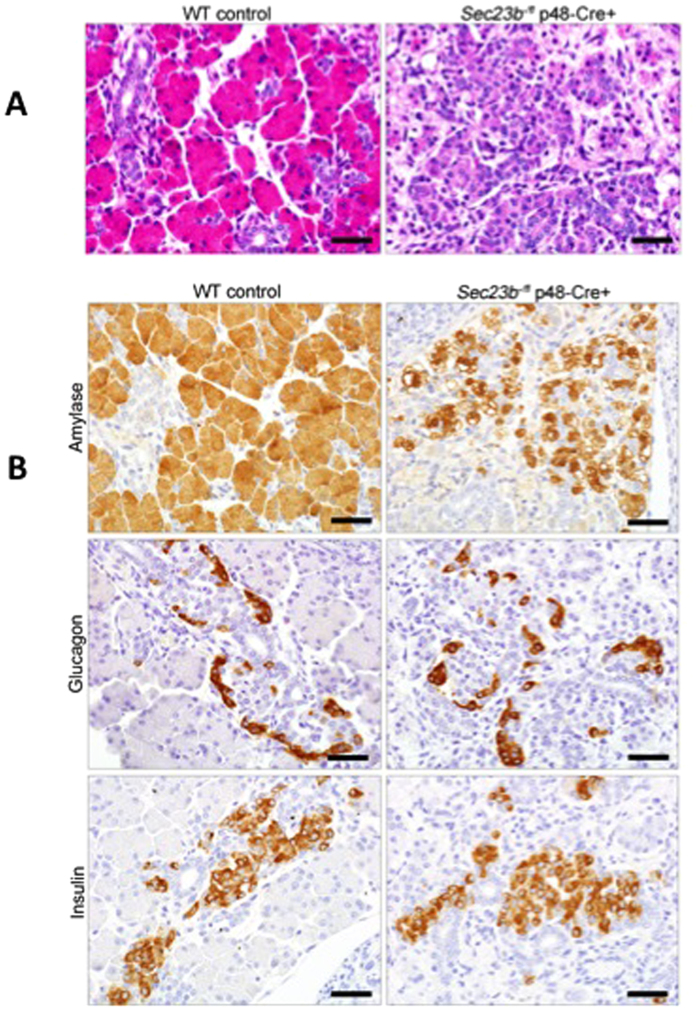
Pancreas tissues obtained from *Sec23b*^−*/fl*^ p48-Cre^+^E18.5 embryos. (**A**) Hematoxilin and eosin staining demonstrates that the pancreata of *Sec23b*^−*/fl*^ p48-Cre^+^ embryos are hypoplastic, with shrunken acini containing small exocrine epithelial cells with scant, often vacuolated, cytoplasms. The ducts and supporting stroma are more prominent in the *Sec23b*^−*/fl*^ p48-Cre^+^ compared to wild type pancreas tissues. In contrast, islets cells appear histologically normal in the *Sec23b*^−*/fl*^ p48-Cre^+^ pancreata. An image is selected from one of six mice evaluated per genotype. (**B**) Immunohistochemistry for amylase, glucagon, and insulin demonstrate that the pancreatic defect in *Sec23b*^−*/fl*^ p48-Cre^+^ mice appears to be confined to the acinar cells. An image is shown from one of six mice evaluated per genotype. Scale bar indicated 25 μm.

Histologic evaluation of pancreatic tissues harvested from E18.5 *Sec23b*^−*/fl*^ p48-Cre^+^ embryos demonstrated hypoplastic pancreatic remnants with degeneration of pancreatic acini (Fig. 2A, supplementary Table 2, p < 0.0001). Acinar cells were smaller than normal, with scant to minimal eosinophilic cytoplasm that was often vacuolated, and separated by clear space and prominent interlobular stroma. Though pancreatic islets could not be identified by H&E staining due to parenchymal collapse, immunostaining for insulin or glucagon demonstrated normal islet cell morphology, while immunohistochemistry for amylase confirmed loss of acinar cells (Fig. 2B).

### Mice with pancreas-specific SEC23A deficiency survive to adulthood and lack a pancreatic phenotype

**Figure 3:**
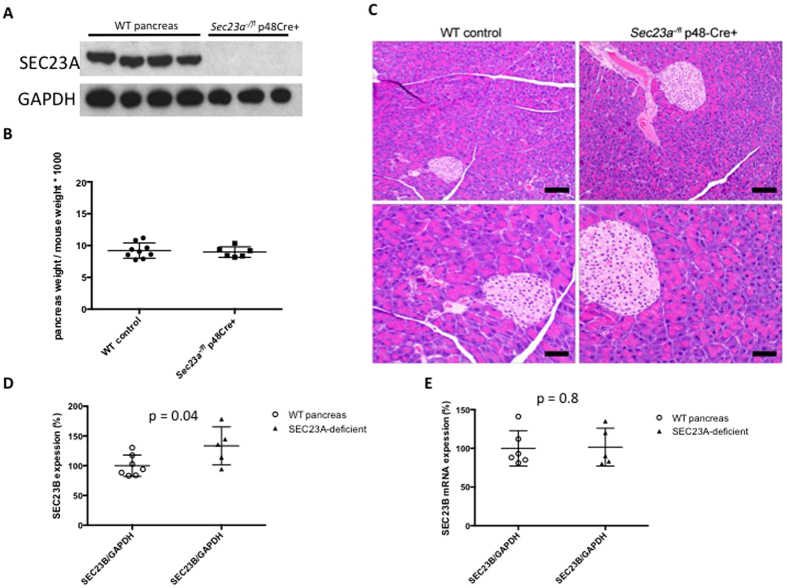
*Sec23a*^−*/fl*^ p48-Cre^+^ mice do not exhibit a pancreatic phenotype. Pancreas tissues harvested from *Sec23a*^−*/fl*^ p48-Cre^+^ mice (**A**) do not have detectable SEC23A protein by western blot, (**B**) have normal weights, (**C**) and appear histologically normal by Hematoxilin and eosin stain (6 mice per genotype were evaluated). Scale bar indicated 50 μm in the top panels and 25 μm in the lower panels. (**D**) SEC23B protein expression is increased by ~33% in SEC23A-deficient pancreata, as measured by quantitative western blots (infrared fluorescent detection). (**E**) SEC23B mRNA expression is indistinguishable in SEC23A-deficient compared to WT pancreata.

Mice homozygous for a *Sec23a* gene trap allele die at mid embryogenesis exhibiting neural tube opening and abnormal development of extra-embryonic membranes^26^. This early lethality precluded the evaluation of the effect of SEC23A deficiency on pancreatic development. To assess the impact of pancreatic SEC23A deficiency on pancreatic development, we generated mice heterozygous for a conditional *Sec23a* floxed allele (*Sec23a*^+*/fl*^) and for a *Sec23a* null allele (*Sec23a*^+/−^) (Fig. 1D–G). Mice with pancreatic deficiency of SEC23A were generated by crossing either *Sec23a*^+*/fl*^ p48-Cre(+) or *Sec23a*^+/−^ *p48-Cre*(+) mice to *Sec23a*^*fl/fl*^ mice. These crosses yielded the expected number of *Sec23a*^*fl/fl*^ *p48-Cre*(+) and *Sec23a*^−*/fl*^ *p48-Cre*(+) offspring (Table 3). *Sec23a*^*fl/fl*^ *p48-Cre*^+^ and *Sec23a*^−*/fl*^ *p48-Cre*^+^ mice exhibited normal survival (mice observed for >300 days of age), development, and fertility. SEC23A protein was undetectable by western blotting in pancreas tissues harvested from *Sec23a*^−*/fl*^ *p48-Cre*^+^ mice (Fig. 3A), consistent with complete excision of the *Sec23a*^*fl*^ allele by the p48Cre-transgene. Pancreas tissues dissected from *Sec23a*^−*/fl*^ *p48-Cre*^+^ mice exhibited normal weights (Fig. 3B) and were histologically indistinguishable from WT controls (Fig. 3C). SEC23B protein demonstrated mildly increased steady state levels in SEC23A-deficient pancreata (Fig. 3D), while SEC23B mRNA was not increased (Fig. 3E). These results demonstrate that SEC23A, in contrast to SEC23B, is dispensable for normal pancreatic development and function.

### No RBC abnormalities observed in mice with erythroid-specific and hematopoietic specific SEC23B deficiency

**Figure 4:**
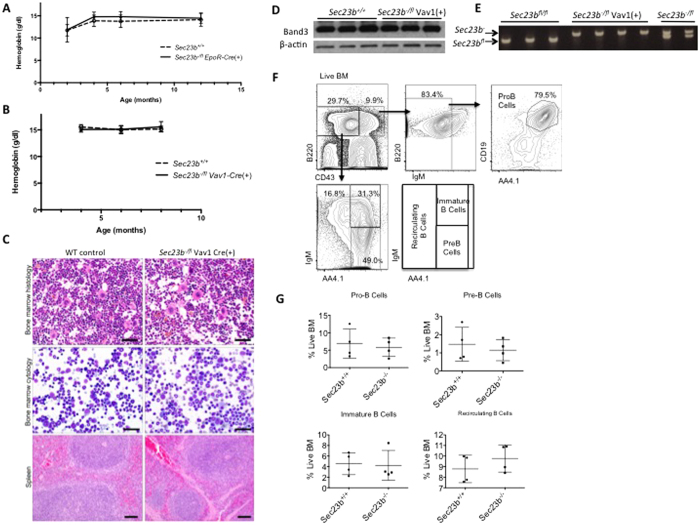
Analysis of the SEC23B deficient bone marrow compartment. (**A,B**) Mice with erythroid-specific (*Sec23b*^−*/fl*^ EpoR-Cre^+^) and pan-hematopoietic (*Sec23b*^−*/fl*^ Vav1-Cre^+^) *Sec23b* deletion do not exhibit anemia, (**C**) increased bi/multi-nucleated erythroid precursors in the bone marrow (scale bars in **C** indicate 25 μm in the top and middle panels, and 50 μm in the lower panels), or (**D**) increased mobility of the RBC membrane protein band 3 by SDS-PAGE. Four mice per genotype we evaluated in Fig. 4**A–C**. (**E**) Genotyping for the *Sec23b*^*fl*^, and *Sec23b*^−^ alleles by PCR, demonstrates complete excision of the floxed allele by Vav1-Cre in the bone marrow compartment. (**F**) Mice transplanted with *Sec23b*^−/−^ or WT control fetal liver cells were evaluated by flow cytometry for a defect in the bone marrow stages of B-lymphocyte development. (**G**) The percentages of pro-B cells, pre-B cells, immature B cells, and recirculating B cells was comparable in bone marrows of mice transplanted with *Sec23b*^−/−^ fetal liver cells and in bone marrows of control mice.

Mice with erythroid-specific or pan-hematopoietic SEC23B-deficiency were generated by crossing the *Sec23b*^*fl*^ allele to mice expressing Cre-recombinase driven by the *EpoR* promoter or the *Vav1* promoter, respectively. *Sec23b*^−*/fl*^ *EpoR-Cre*(+) and *Sec23b*^−*/fl*^ *Vav1-Cre*(+) mice were observed in the expected Mendelian ratios at weaning (Table 2), appeared grossly normal, and exhibited normal survival and fertility, as well as normal RBCs, with none of the features of human CDAII. Specifically, these mice exhibited no anemia (Fig. 4A,B), no increased bi/multi-nucleated erythroid precursors in the bone marrow (Fig. 4C), and no increased mobility of band 3 on SDS PAGE (Fig. 4D). *Vav1-Cre* mediated excision of the *Sec23b* floxed allele appeared complete in bone marrow cells (Fig. 4E).

### Murine hematopoietic SEC23B deficiency does not result in a block of B-lymphocyte development

In a previous report^24^, a subtle B-cell defect was suggested in cells derived from SEC23B-deficient hematopoietic precursors. To further characterize the B-lineage development in SEC23B null mice, C57BL/6 J mice ubiquitously expressing GFP (UBC-GFP^tg/+^) were lethally irradiated and transplanted with fetal liver cells (FLC) isolated from E16.5 *Sec23b*^−/−^ or WT embryos. Eight weeks post-transplantation, reconstituted BM hematopoietic cells were GFP(−), confirming donor HSC engraftment. The percentages of GFP(−) BM pro-B cells, pre-B cells, recirculating B cells, and immature B cells were equivalent in mice transplanted with *Sec23b*^−/−^ FLC and mice transplanted with WT FLC (Fig. 4F,G), thereby arguing against a major defect in B-lymphocyte development resulting from SEC23B deficiency.

## Discussion

In humans, homozygous or compound heterozygous loss of function mutations in *SEC23B* result in a phenotype limited to the erythroid compartment, with no other non-hematologic abnormalities reported. In contrast, mice homozygous for a *Sec23b* genetrap allele die perinatally exhibiting extensive pancreatic degeneration, and mice with hematopoietic deficiency for SEC23B exhibit no RBC phenotype.

In this report, we confirm the perinatal mortality and pancreatic phenotype of SEC23B deficient mice using an independent *Sec23b* null allele (*Sec23b*^−^). We also show that two independent BAC transgenes spanning *Sec23b* rescue the lethality and pancreatic phenotype of *Sec23b*^*gt/gt*^ mice, thereby confirming that the latter phenotype observed in these mice is indeed a result of SEC23B deficiency and definitively excluding an off-target genetrap effect on a nearby gene (passenger gene mutation) segregating with the *Sec23bgt* allele as a cause of the phenotype. We also show that mice with either erythroid specific or pan-hematopoietic *Sec23b* deletion do not exhibit a CDAII phenotype. These data are consistent with previously reported HSC transplantation results^24^, and exclude an artifact from HSC transplantation in our prior report as the cause of the lack of RBC phenotype in mice transplanted with SEC23B deficient HSCs^24^. Furthermore, we demonstrate that the stages of B-lymphocyte development in the BMs of mice transplanted with SEC23B deficient HSCs are indistinguishable from those in BMs of control mice transplanted with wild type HSCs, thereby ruling out a defect in B-lymphocyte development resulting from SEC23B deficiency, as previously suggested^24^.

We also show that mice with pancreatic SEC23A deficiency survive normally and are indistinguishable from their wild type littermate controls, exhibiting normal pancreas weight and histology. These data demonstrate that SEC23B, but not SEC23A, is essential for murine pancreatic development. SEC23B protein but not SEC23B mRNA was increased in SEC23A-deficient pancreata. These data suggest that relative SEC23A and SEC23B protein levels may be regulated in part by competition for SEC24 subunits^24^, with increased stability of the SEC23 subunit within a SEC23-SEC24 heterodimer.

We show that deletion of *Sec23b* exclusively in the pancreas is sufficient to account for the lethality of mice with germline deficiency for SEC23B. These data exclude a previously unidentified pathology, ectopic to the pancreas, as a cause of the pancreatic phenotype and suggest that the corresponding pancreatic destruction is due to a cell-autonomous defect in the pancreatic cell itself. Immunohistochemical stains demonstrate morphologically abnormal acinar cells but normal islet cells. These data suggest that the observed pancreatic degeneration is a result of an acinar cell defect, potentially due to delayed ER exit of a specific secretory cargo(s), possibly one or more exocrine pancreatic enzymes, which when retained in the ER results in pancreatic degeneration.

The rescue of the SEC23B deficiency phenotype by either of two BAC transgenes demonstrates that the regulatory elements critical for physiologic pancreatic expression of *Sec23b* reside within the minimum region shared by the BAC transgenes. These findings have important implications for future work aiming at defining therapeutic approaches to modifying the expression of the *SEC23* paralogs in CDAII and other *SEC23* disorders^17^,^18^,^23^.

## Materials and Methods

### Generation of *Sec23b* mutant mouse lines

Two independent *Sec23b* mutant mouse lines, one with a gene trap insertion in intron 19 of the gene (*Sec23b*^*gt*^), and another line with a conditional gene trap (flanked by *FRT* sites) insertion in intron 4 of *Sec23b (Sec23b*^*cgt*^) were previously described^22^,^23^ (Fig. 1A,C). Mice expressing FLP recombinase driven by the human *β-actin* promoter (*β-actin FLP*) (Jackson laboratory stock # 005703) were crossed to the *Sec23b*^*cgt*^ allele to excise the gene trap cassette, resulting in the *Sec23b* floxed allele (*Sec23b*^*fl*^), with exons 5 and 6 flanked by *loxP* sites. The *Sec23b*^*fl*^ allele was crossed to mice ubiquitously expressing Cre recombinase under the control of an *EIIa* promoter (*EIIa-Cre*) (Jackson laboratory stock # 003724) to excise exons 5 and 6 and generate a null *Sec23b* allele (*Sec23b*^−^), resulting in a frameshift and early stop codon in exon 7 (Fig. 1C). *Sec23b*^+/−^ mice were back-crossed with C57BL/6 J mice to remove the *EIIA-Cre* transgene. Mice with a pancreas-specific SEC23B knock-out were generated by crossing *Sec23b*^*fl/fl*^ or *Sec23b*^−*/fl*^ mice to mice expressing Cre recombinase driven by either the *p48* promoter (generous gift from Dr. Christopher Wright)^27^ or the *Pdx1* promoter^28^. Mice with erythroid-specific and pan-hematopoietic SEC23B-deficiency were generated by crossing the *Sec23b*^*fl*^ allele to *EpoR-Cre* mice^29^ (generous gift from Dr. Ursula Klingmüller) and *Vav1-Cre* mice (Jackson laboratory stock # 008610), respectively. *Sec23b*^*gt*^ mice used in this study were backcrossed to C57BL/6 J mice for >10 generations. The *Sec23b*^*cgt*^ allele was derived from a C57BL/6 ES cell, and the *Sec23b*^*fl*^ and *Sec23b*^−^ alleles were maintained on a pure C57BL/6 J background. All Cre mice were back-crossed to C57BL/6 J mice for >6 generations. Mice were housed at the Life Sciences Institute of the University of Michigan, and all experiments were approved by and performed in accordance with the regulations of the University Committee on Use and Care of Animals.

### Generation of a *Sec23a* conditional mutant mouse line

Mice heterozygous for a *Sec23a* conditional gene trap (flanked by *FRT* sites) insertion into intron 2 of *Sec23a (Sec23a*^+*/cgt*^) were derived from an embryonic stem (ES) cell clone (EPD0072_1_B09) and live mice were obtained from the Knock-Out Mouse Project (KOMP) Repository. The *Sec23a*^*cgt*^allele was derived from C57BL/6 ES cells and subsequently maintained on a pure C57BL/6 J background. *Sec23a*^+*/cgt*^mice were crossed to *β-actin FLP* mice to excise the gene-trap cassette, resulting in the *Sec23a*^*fl*^ allele, with exon 3 flanked by *LoxP* sites (Fig. 1D). *Sec23a*^+*/fl*^ mice were back-crossed with C57BL/6 J mice to remove the *FLPe* transgene. *Sec23a*^+*/fl*^ mice were crossed to *EIIA-Cre* transgenic mice to excise exon 3, resulting in a null *Sec23a* allele (*Sec23a*^−^) with a frameshift and an early stop codon. *Sec23a*^+/−^ mice were back-crossed with C57BL/6 J mice to remove the *EIIA-Cre* transgene. Mice with pancreas-specific SEC23A deficiency were generated by crossing the *Sec23a*^*fl*^ allele to *p48-Cre*, as above.

### Generation of BAC transgenic mice

Two bacterial artificial chromosome (BAC) clones spanning the *Sec23b* gene, RP23-70J9 (RP23) and RP24-371A4 (RP24), were purchased from the BACPAC Resources Center at Children’s Hospital Oakland Research Institute. BAC DNA constructs were expanded in One Shot TOP10 *Escherichia coli* and purified using the NucleoBond BAC100 kit (Machery-Nagel). BAC DNA was injected into zygotes generated from a cross between C57BL/6JxSJL F1 females and *Sec23b*^+*/*+^ male mice. RP23 and RP24 transgenic founders (*Sec23b*^+*/*+^Tg^+^) were crossed to *Sec23b*^+*/gt*^ mice, and the *Sec23b*^+*/gt*^ Tg^+^ progeny were crossed to *Sec23b*^+*/gt*^ C57BL/6 J mice to generate potential *Sec23b*^*gt/gt*^ Tg^+^ “rescue mice”. Mice were genotyped for the *Sec23b* allele and for the presence of the BAC transgene. Standard genotyping methods are unable to differentiate between the endogenous *Sec23b* allele and the *Sec23b* gene present on the BAC transgene. Therefore, microsatellite genotyping was used (see below) to distinguish *Sec23b*^+*/gt*^ Tg^+^ mice from *Sec23b*^*gt/gt*^ Tg^+^ mice.

### PCR genotyping

DNA was isolated from mouse tail biopsies and genotyping was performed using the Go-Taq Green Master Mix (Promega). Genotyping for the *Sec23b*^*cgt*^, *Sec23b*^*fl*^, and *Sec23b*^−^ alleles and for the various Cre transgenes was performed as previously described^24^,^27^,^28^. Location of the *Sec23a* genotyping primers is shown in Fig. 2A, and their sequences are shown in Supplementary Table 1. The *Sec23a* cgt allele was genotyped in a three-primer PCR assay using a forward primer (primer A) located in *Sec23a* intron 2, upstream of the gene trap insertion site and two reverse primers, one (primer B) located in the gene trap insertion cassette between the two FRT sites and the second (primer B4) located in intron 2 downstream of primer A (the genomic sequence to which primer B4 anneals is absent from *Sec23a*^*cgt*^). This PCR product was resolved by 2% (weight/volume) agarose gel electrophoresis (Fig. 1E). Genotyping for the *Sec23a*^*fl*^ allele was performed with a PCR assay consisting of the forward primer A and a reverse primer located in intron 2 between the two *LoxP* sites (primer E2) (Fig. 1F). This reaction does not yield a PCR product for the *Sec23a*^*−*^ allele. Confirmation of the excision of exon 3 (*Sec23a*^−^ allele) was performed using a PCR assay with primer A and a reverse primer located in intron 3 downstream of the *LoxP* site (primer D) (Fig. 1G).

### Microsatellite genotyping

To distinguish *Sec23b*^*gt/gt*^ Tg^+^ from *Sec23b*^+*/gt*^ Tg^+^ mice, a microsatellite genotyping assay was designed that differentiates the endogenous *Sec23b* wild type allele from the *Sec23b* Tg^+^ allele originally targeted on the 129/SvImJ background. The gene trap is expected to be 129/SvImJ within the congenic interval, in contrast to the wild-type allele, which should be either C57BL/6 J or SJL/J (SJL/J was introduced with the transgenic founders). A similar microsatellite genotyping strategy was previously described^30^. Microsatellites were selected using the Tandem Repeat Database^31^. Microsatellite genotyping was performed on potential *Sec23b*^*gt/gt*^ Tg^+^ mice by PCR on genomic DNA, using the colorless GoTaq Hot Start Master Mix (Promega) with a forward primer (MS-F) located upstream of the microsatellite and a reverse primer (MS-R) downstream of the microsatellite. PCR products were separated on the Caliper Labchip 90 Instrument using the HT DNA Chip for automated PCR product size determination, according to the manufacturer’s instructions.

### Fetal Liver cells transplants

Timed matings were performed by intercrossing *Sec23b*^+/−^ mice as previously described^24^. Pregnant females were euthanized at E16.5 postcoitus, and fetal liver cells were isolated and transplanted into lethally irradiated recipient mice as previously described^24^.

### Complete blood counts (CBC) and bone marrow (BM) analysis

Blood (20 or 70 microliters) was collected from the retro-orbital venous sinuses of anesthetized mice via anticoagulant-coated capillary tubes. Blood was diluted in 5% bovine serum albumin in phosphate buffered saline (pH 7.4) to a total volume of 200 microliters. CBCs were determined as previously described^24^.

BM cells were collected from hind limbs of euthanized mice, cytospins prepared and stained as previously described^24^, and the latter examined under light microscopy by an investigator blinded to the BM genotype.

### Flow cytometry and RBC ghost preparation

BM cells were stained with the following antibodies obtained from BioLegend: anti-B220, anti-CD19, anti-CD43, anti-CD93 (AA4.1), and anti-sIgM. Analysis was performed using a FACSAria III (Becton Dickinson Biosciences). Non-viable cells were excluded with 4′6-diamidino-2-phenylindole (Sigma). Files were analyzed with FlowJo (Tree Star).

RBC ghosts were prepared from peripheral blood RBC and stored at −80 °C in lysis buffer, as previously described^24^.

### Western blot and qRT-PCR

Total cell lysates were prepared as previously described^24^. Western blots (film visualization with chemiluminescent detection) and quantitative western blot (Infrared fluorescent detection) were performed as previously described^22^. For quantitative western blots, band intensities were quantified using the Image Studio software (LI-COR Biosciences) and normalized to GAPDH, and the secondary antibodies utilized were IRDye 680RD or IRDye 800CW. qRT PCR was performed as previously described^24^.

### Antibodies

Rabbit Anti-mouse SEC23B and anti-mouse SEC23A antibodies were generated as previously described^24^,^25^. Mouse anti-GAPDH and anti-Band3 antibodies were purchased from Millipore. Anti-Actin antibody was purchased from Santa Cruz.

### Hematoxylin and eosin staining and Immunohistochemistry

At necropsy, tissues were collected and fixed in aqueous buffered zinc formalin (Z-fix, Anatech) for histologic and immunohistochemical analysis. Tissues were routinely processed, embedded in paraffin, sectioned at 4 um, and stained with hematoxylin and eosin (H&E). For immunohistochemistry, rabbit polyclonal antibodies to pancreatic amylase (ab21156, 1:1000; Abcam, Cambridge MA), insulin (C27C9, 1:800; Cell Signaling Technology, Danvers MA), and glucagon (D16G10, 1:100; Cell Signaling Technology, Danvers MA) were used. Following antigen retrieval, quenching of endogenous peroxidases and rodent block, primary antibodies were applied. After primary antibody incubation and washing, rabbit polymer HRP secondary antibody (Biocare, Concord CA), was applied. Negative controls were obtained by substitution of the primary antibody with Universal Negative reagent (Biocare, Concord CA). Following washing, 3,3-diaminobenzidine (DAB) was applied to visualize all reactions. Sections were counterstained with hematoxylin, dehydrated through graded alcohols, immersed in xylene, and mounted with coverslips. Histologic evaluation was performed by an investigator blinded to the genotype of the evaluated mice.

### Statistical analysis

The Chi-square test was used to calculate the statistical significance of the deviation of the genotypes of a given cross from the expected Mendelian ratios. The statistical significant differences between blood count parameters in test cohorts and controls were calculated using the student’s T-test.

## Additional Information

**How to cite this article**: Khoriaty, R. *et al*. Pancreatic SEC23B deficiency is sufficient to explain the perinatal lethality of germline SEC23B deficiency in mice. *Sci. Rep.* **6**, 27802; doi: 10.1038/srep27802 (2016).

## Supplementary Material

Supplementary Information

## Figures and Tables

**Table 1:** Rescue of *Sec23b^gt/gt^* by BAC transgenes and results of *Sec23b*^+/−^ crosses.

A. Genotype:	*Sec23b*^*gt/gt*^ Tg^+^	Other	−	*p*-value*
***Sec23b***^**+*****/gt***^ **x** ***Sec23b***^**+*****/gt***^ **Tg**^**+**^ **Expected ratios**	**14.3% (1/7)**	**85.7% (6/7)**	−	
Total for RP23 BAC (n = 102)	8.8% (n = 9)	91.2% (n = 93)	−	>0.6
Total for RP24 BAC (n = 95)	10.5% (n = 10)	89.5% (n = 85)	−	>0.7
**B. Genotype:**	***Sec23b***^**+*****/*****+**^	***Sec23b***^+/−^	***Sec23b***^−/−^	**p-value**^**#**^
***Sec23b***^+/−^ **x** ***Sec23b***^+/−^ **Expected ratios**	**25%**	**50%**	**25%**	
Observed at weaning % (n = 95)	33% (31)	67% (64)	0% (0)	<0.0001
Observed at E.18.5% (n = 31)	35% (11)	42% (13)	22.6% (7)	>0.7
Observed at E.17.5% (n = 55)	22% (12)	53% (29)	25% (14)	>0.9
***Sec23b***^+/−^ **x** ***Sec23b***^**+*****/*****+**^ **Expected ratios**	**50%**	**50%**	−	
Observed at weaning % (n = 204)	51% (105)	49% (99)	−	>0.84

(**A**) Rescue of *Sec23b*^*gt/gt*^ by BAC transgenes. Genotypes were performed at weaning. *P-value calculated for *Sec23b*^*gt/gt*^Tg^+^ versus all other genotypes. (**B**) Results of *Sec23*^+/−^ intercrosses and of *Sec23b*^+/−^ backcrosses with wild type C57BL/6J mice. ^#^P-value calculated for *Sec23b*^−/−^ versus all other genotypes, except in the *Sec23b*^+/−^ x *Sec23b*^+*/*+^ cross, where P-value is calculated for *Sec23b*^+/−^ versus *Sec23b*^+*/*+^ mice.

**Table 2:** Results of matings to generate mice with pancreas specific *Sec23b* deletion using *Pdx-Cre* (A) and *p48-Cre* (B), and to generate mice with erythroid specific (C) or pan-hematopoietic (D) *Sec23b* deletion using *EpoR-Cre* or *Vav1-Cre*, respectively.

A. Genotypes:	*Sec23b*^+*/*+^*Pdx-Cre*(+)	*Sec23b*^+*/*+^*Pdx-Cre* (−)	*Sec23b*^+*/fl*^*Pdx-Cre*(+)	*Sec23b*^+*/fl*^*Pdx-Cre*(−)	*Sec23b*^+/−^*Pdx-Cre*(+)	*Sec23b*^+/−^*Pdx-Cre*(−)	*Sec23b*^−*/fl*^*Pdx-Cre*(+)	*Sec23b*^−*/fl*^*Pdx-Cre*(−)	p-value^*^
***Sec23b***^**+*****/fl***^ **Pdx-Cre(+) x** ***Sec23b***^+/−^ **Expected ratios**	**12.5%**	**12.5%**	**12.5%**	**12.5%**	**12.5%**	**12.5%**	**12.5%**	**12.5%**	
Observed at weaning % (n = 74)	16% (12)	11% (8)	16% (12)	11% (8)	16% (12)	12% (9)	3% (2)	15% (11)	<0.016
**B. Genotypes:**	***Sec23b***^**+*****/*****+**^ ***p48-Cre*****(+)**	***Sec23b***^**+*****/*****+**^ ***p48-Cre***(−)	***Sec23b***^**+*****/fl***^ ***p48-Cre*****(+)**	***Sec23b***^**+*****/fl***^ ***p48-Cre***(−)	***Sec23b***^+/−^ ***p48-Cre*****(+)**	***Sec23b***^+/−^ ***p48-Cre***(−)	***Sec23b***^**−*****/fl***^ ***p48-Cre*****(+)**	***Sec23b***^**−*****/fl***^ ***p48-Cre***(−)	**p-value**^**#**^
***Sec23b***^**+*****/fl***^ **p48-Cre(+) x** ***Sec23b***^+/−^ **Expected ratios**	**12.5%**	**12.5%**	**12.5%**	**12.5%**	**12.5%**	**12.5%**	**12.5%**	**12.5%**	
Observed at weaning % (n = 148)	17% (25)	11% (17)	12% (18)	17% (25)	14% (21)	14% (20)	0% (0)	15% (22)	<0.0001
Observed at E.18.5% (n = 52)	10% (5)	8% (4)	13% (7)	12% (6)	17% (9)	13% (7)	15% (8)	12% (6)	>0.5
Postnatal death within 1 day of birth (n = 4)	0% (0)	0% (0)	0% (0)	0% (0)	0% (0)	0% (0)	100% (4)	0% (0)	<0.0001
**C. Genotypes:**	***Sec23b***^**+*****/*****+**^ ***EpoR-Cre*****(+)**	***Sec23b***^**+*****/*****+**^ ***EpoR- Cre***(−)	***Sec23b***^**+*****/fl***^ ***EpoR-Cre*****(+)**	***Sec23b***^**+*****/fl***^ ***EpoR-Cre***(−)	***Sec23b***^+/−^ ***EpoR-Cre*****(+)**	***Sec23b***^+/−^ ***EpoR-Cre***(−)	***Sec23b***^**−*****/fl***^ ***EpoR-Cre*****(+)**	***Sec23b***^**−*****/fl***^ **EpoR-Cre**(−)	**p-value^**
***Sec23b***^**+*****/fl***^ **x** ***Sec23b***^+/−^ **EpoR-Cre(+) Expected ratios**	**12.5%**	**12.5%**	**12.5%**	**12.5%**	**12.5%**	**12.5%**	**12.5%**	**12.5%**	
Observed at weaning % (n = 80)	12% (10)	10% (8)	10% (8)	16% (13)	10% (8)	13% (10)	15% (12)	14% (11)	>0.7
**D. Genotypes:**	***Sec23b***^**+*****/fl***^ ***Vav1-Cre*****(+)**	***Sec23b***^**+*****/fl***^ ***Vav1-Cre***(−)	***Sec23b***^**−*****/fl***^ ***Vav1-Cre*****(+)**	***Sec23b***^**−*****/fl***^ ***Vav1-Cre***(−)	−	−	−	−	**p-value**^**$**^
***Sec23b***^***fl/fl***^ **x** ***Sec23b***^+/−^ **Vav1-Cre(+) Expected ratios**	**25%**	**25%**	**25%**	**25%**	−	−	−	−	
Observed at weaning % (n = 43)	21% (9)	19% (8)	28% (12)	32% (14)	−	−	−	−	>0.4

^*^p-value calculated for *Sec23b*^−*/fl*^*Pdx-Cre*(+) versus all other genotypes. ^#^P-value calculated for *Sec23b*^−*/fl*^*p48-Cre*(+) versus all other genotypes. ^P-value calculated for *Sec23b*^−*/fl*^*EpoR-Cre*(+) versus all other genotypes. ^$^P-value calculated for *Sec23b*^−*/fl*^*Vav1-Cre*(+) versus all other genotypes.

**Table 3:** Results of matings to generate mice with pancreas specific *Sec23a* deletion, using p48-Cre.

Genotypes:	*Sec23a*^+*/fl*^*p48-Cre*(+)	*Sec23a*^+*/fl*^*p48-Cre*(−)	*Sec23a*^*fl/fl*^*p48-Cre*(+)	*Sec23a*^*fl/fl*^*p48-Cre*(−)	p-value^#^
***Sec23a***^**+*****/fl***^ ***p48-Cre*****(+) x** ***Sec23a***^***fl/fl***^ **Expected ratios**	25%	25%	25%	25%	
Observed at weaning % (n = 33)	36.4% (12)	27.3% (9)	15.1% (5)	21.2% (7)	>0.6
**Genotypes:**	***Sec23a***^**+*****/fl***^***p48-Cre*****(+)**	***Sec23a***^**+*****/fl***^***p48-Cre***(−)	***Sec23a***^**−*****/fl***^***p48-Cre*****(+)**	***Sec23a***^**−*****/fl***^***p48-Cre***(−)	**p-value**^**#**^
***Sec23a***^+/−^ ***p48-Cre*****(+) x** ***Sec23a***^***fl/fl***^ **Expected ratios**	**25%**	**25%**	**25%**	**25%**	
Observed at weaning % (n = 29)	34.4% (10)	27.6% (8)	20.7% (6)	17.3% (5)	>0.7

^#^P-value calculated for *Sec23a*^−*/fl*^*p48-Cre*(+) or *Sec23a*^−*/fl*^*p48-Cre*(+) mice versus all other genotype.
