# Supplementary material for: Pancreatic SEC23B deficiency is sufficient to explain the perinatal lethality of germline SEC23B deficiency in mice
Source: Sci Rep. 2016 Jun 14;6:27802. doi: 10.1038/srep27802 (PMC4906273; doi:10.1038/srep27802)
Supplement: Supplementary Information [file srep27802-s1.doc]

**Supplementary data**

Title: Pancreatic SEC23B deficiency is sufficient to explain the perinatal lethality of germline SEC23B deficiency in mice

Running title: Pancreas specific SEC23B and SEC23A deletion

Rami Khoriaty, Lesley Everett, Jennifer Chase, Guojing Zhu, Mark Hoenerhoff, Brooke McKnight, Matthew P. Vasievich, Bin Zhang, Kärt Tomberg, John Williams, Ivan Maillard, David Ginsburg

| **Primer** | **Primer Sequence** |
| --- | --- |
| Primer A | GGAGCTGTTCCAAGCACATTCTCT |
| Primer B | CACAACGGGTTCTTCTGTTAGTCC |
| Primer B4 | CAGAGCCATGGTAGAAGAGATCCAA |
| Primer E2 | TCCCTGGGTCAAAGTGCTGTCCA |
| Primer D | CCACGCAGTCCTGGCTCTCCTGA |
| MS-F | GCCTCCATCTGCTTACAAGG |
| MS-R | TTGGATTGAATTGCTGTGGA |

Supplementary table1. Primer sequences


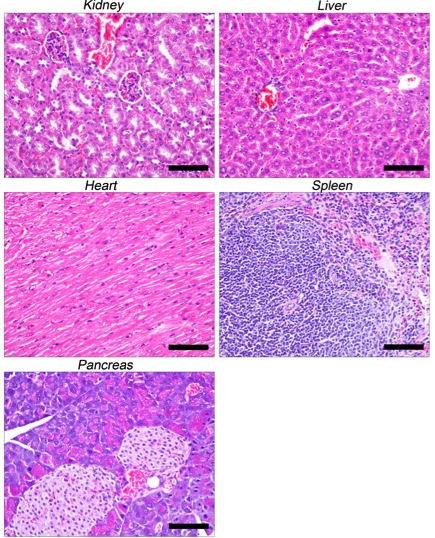


**Supplementary Figure 1.** Tissues harvested from Sec23b^gt/gt^ Tg^+^ mice exhibited normal histology. Heart, kidney, liver, pancreas, and spleen histology are shown in this figure. An image is shown from 3 mice evaluated. Scale bar indicated 50 μm.

**
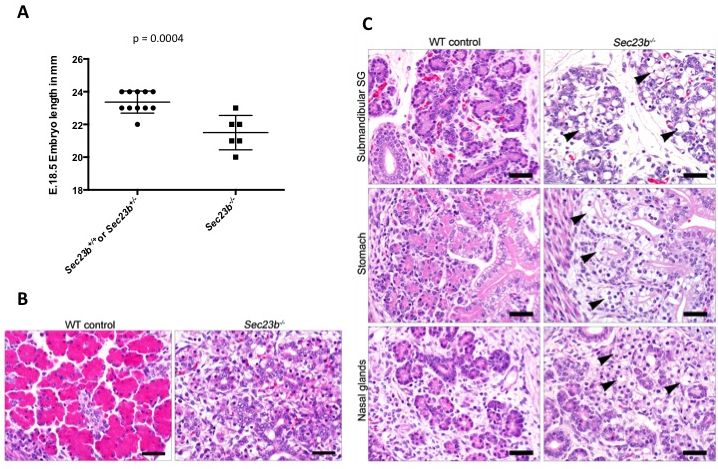
**

**Supplementary Figure 2. Histology of embryos ubiquitously deficient in SEC23B.** (A) *Sec23b^-/-^* embryos are smaller in length than their littermate controls (*Sec23b^+/+^* and *Sec23b^+/-^* embryos had similar lengths and were grouped together). (B) *Sec23b^-/-^* embryos exhibit hypoplastic pancreata, characterized by a general lack of exocrine lobules, with mild decrease acinar cell volume and absence of defined zymogen granules. The stroma was prominent and pancreatic ducts appeared relatively unaffected (n = 4 mice per genotype). (C) Vacuolar and hypoplastic lesions were also apparant in other glandular tissues including the salivary glands, stomach, and nasal glands. The salivary glands changes were characterized by cytoplasmic vacuolation (arrowheads) and variably sized glandular epithelial cells with decreased cytoplasm or eosinophilic tincture. In the stomach, there was similar vacuolar changes (arrowheads) to glandular epithelial cells within gastric pits, with relative sparing of superficial gastric epithelium. Cytoplasmic vacuolation (arrowheads) and glandular hypoplasia were observed in nasal glands. The cytoplasmic vacuolation is non-specific and could indicate a derangement in normal cellular function, apoptosis, or autophagy. An image is shown from 4 mice per genotype evaluated. Scale bar indicated 25 μm.


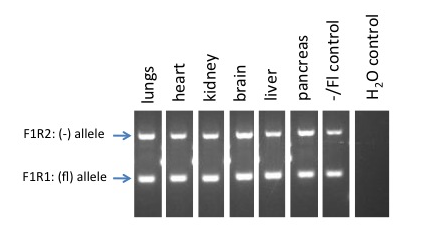


**Supplementary Figure 3. Genotyping for *Sec23b* in tissues harvested from the 2 *Sec23b^-/fl^* Pdx-Cre^+^ mice that survived to adulthood.** A PCR genotyping assay demonstrated a persistent high-level detection of the *Sec23b^fl^* allele in the pancreatic tissue of both surviving mice (comparable to other tissues), explaining the survival of these mice into adulthood.

| WT control | *Sec23b^-/fl^* p48 Cre (+) |
| --- | --- |
| 2/200 | 200/200 |
| 0/200 | 200/200 |
| 1/200 | 200/200 |
| 1/200 | 200/200 |

Supplementary table 2. Number of histologically abnormal cells (see figure 2A for description) divided by total number of acinar cells evaluated. Pancreas tissues from 4 difference mice were evaluated per each genotype (p-value < 0.0001).
